# Promising Assays for Examining a Putative Role of Ribosomal Heterogeneity in COVID-19 Susceptibility and Severity

**DOI:** 10.3390/life12020203

**Published:** 2022-01-28

**Authors:** Yih-Horng Shiao

**Affiliations:** US Patent Trademark Office, Department of Commerce, Alexandria, VA 22314, USA; yihhorng@aol.com

**Keywords:** translation machinery, SARS-CoV-2, COVID-19, ribosomes, ribosomal heterogeneity, ribosomal RNAs, ribosomal proteins, assays for RNA sequence and structure, assays for proteins

## Abstract

The heterogeneity of ribosomes, characterized by structural variations, arises from differences in types, numbers, and/or post-translational modifications of participating ribosomal proteins (RPs), ribosomal RNAs (rRNAs) sequence variants plus post-transcriptional modifications, and additional molecules essential for forming a translational machinery. The ribosomal heterogeneity within an individual organism or a single cell leads to preferential translations of selected messenger RNA (mRNA) transcripts over others, especially in response to environmental cues. The role of ribosomal heterogeneity in SARS-CoV-2 coronavirus infection, propagation, related symptoms, or vaccine responses is not known, and a technique to examine these has not yet been developed. Tools to detect ribosomal heterogeneity or to profile translating mRNAs independently cannot identify unique or specialized ribosome(s) along with corresponding mRNA substrate(s). Concurrent characterizations of RPs and/or rRNAs with mRNA substrate from a single ribosome would be critical to decipher the putative role of ribosomal heterogeneity in the COVID-19 disease, caused by the SARS-CoV-2, which hijacks the host ribosome to preferentially translate its RNA genome. Such a protocol should be able to provide a high-throughput screening of clinical samples in a large population that would reach a statistical power for determining the impact of a specialized ribosome to specific characteristics of the disease. These characteristics may include host susceptibility, viral infectivity and transmissibility, severity of symptoms, antiviral treatment responses, and vaccine immunogenicity including its side effect and efficacy. In this study, several state-of-the-art techniques, in particular, chemical probing of ribosomal components or rRNA structures, proximity ligation to generate rRNA-mRNA chimeras for sequencing, nanopore gating of individual ribosomes, nanopore RNA sequencing and/or structural analyses, single-ribosome mass spectrometry, and microfluidic droplets for separating ribosomes or indexing rRNAs/mRNAs, are discussed. The key elements for further improvement and proper integration of the above techniques to potentially arrive at a high-throughput protocol for examining individual ribosomes and their mRNA substrates in a clinical setting are also presented.

## 1. Interaction of SARS-CoV-2 with Ribosome

The severe acute respiratory syndrome-coronavirus-2 (SARS-CoV-2) is a single-stranded RNA virus and relies on host translational machinery to synthesize non-structural proteins (NSP1 to NSP16) and structural proteins (envelope, spike, membrane, and nucleocapsid). This allows the virus to prioritize its life cycle by evading and disrupting the host defense mechanisms [1,2,3]. Interactions of viral proteins and RNAs with host ribosomal translational machinery have been extensively characterized in cultured cells by chemical and/or physical cross-linking strategies followed by mass spectrometry and/or RNA-sequencing. One such study showed that NSP1 binds to a helix 18 region of the 18S ribosomal RNA (rRNA) including the G626 nucleotide near the messenger RNA (mRNA) entry channel. This potentially blocks the 40S ribosomal subunit to scan host mRNA and the recruitment of transfer RNAs (tRNAs) to the active 80S ribosome. Conversely, NSP8 attacks the 28S rRNA at the expansion segment ES27 of the 60S ribosomal subunit and may interfere with the exit of newly translated protein [4]. NSP1 upon binding to the 40S subunit also accelerates degradation of cellular mRNAs but protects viral RNAs containing a 5′-leader that is added during negative-strand synthesis [5]. In addition, NSP1 modulates ribosomal preinitiation complex, which contains eukaryotic translation initiation factors (EIFs) and the initiator tRNA to refrain cap-dependent host mRNAs from binding to the 40S subunit [6]. Viral RNA physically interacts with translational machinery, including several ribosomal proteins of the cytosolic 80S ribosome, translation initiation factors (such as EIF3B, 4H, 4B, 3F, and A3), and mitochondrial 12S and 16S rRNAs [7]. These and other ribosomal components of the small 40S or large 50S ribosome subunit, translation initiation complex, and translation elongation factor have been also consistently detected to form complexes with SARS-CoV-2 RNA [8,9,10].

Selected mutations of the SARS-CoV-2 RNA have been shown to influence the efficacy of programmed -1 ribosomal frameshift via pseudoknot structure that determines the final production of either viral pp1a or pp1ab polypeptide from the corresponding open reading frame 1a (ORF1a) or ORF1b [11]. Such pseudoknot is embedded within a frameshift element-arch as a result of a ≈1.5-kb long RNA-RNA interaction of the viral genome [12]. The pseudoknot-mediated programmed -1 ribosomal frameshift activity also depends on the proper folding of the viral genome, which is supported by a computational prediction of 98 co-variant base pairs out of 200,621 SARS-CoV-2 strains in comparison with reference sequence [13]. This suggests that the efficiency of frameshift may be diverse among viral strains. Moreover, those mechanisms of direct interactions with ribosomes, extracellular binding of spike protein to ACE2 receptor has been shown to trigger phosphorylation and release of the ribosomal L13a protein from active translating ribosome [14]. SARS-CoV-2 also stoichiometrically reduces the cellular RNA-binding proteins, including EIF3 (D, E, and L) and EIF2S1 in infected cells [15]. Proper co-translational folding of the RNA-dependent RNA polymerase NSP12, mediated by pausing of ribosome at rare codons, is essential for producing active enzyme. It may have an implication for the natural selection of SARS-CoV-2 variants [16].

## 2. Heterogeneity of Ribosome

One direct evidence of ribosomal heterogeneity comes from ribosomopathy, caused by defective RPs and/or rRNAs. In the Diamond-Blackfan Anemia (DBA), ribosomes with deficient ribosomal protein RPS26 preferentially translate mRNAs from stress-response pathway while those having proficient RPS26 recognize mRNAs with defined Kozak sequence [17]. In addition, the RPL5 genetic variant rs376208311 showed less calculated ribosomal loads compared with wild-type sequence, presumably leading to stoichiometric reduction of RPL5 protein in the ribosomes and DBA cells [18]. In mouse embryonic stem cells, a lower abundance of several ribosomal proteins in polysomes compared with free nonactive ribosomes was detected. Further ribosomal profiling analysis revealed that ribosomes categorized by the presence or absence of tagged RPL10A or RPS25 display differential binding preference to distinct sets of transcripts [19]. Using such approach of tagging ribosomal proteins endogenously, several ribosome-associated proteins and preferential transcript substrates also have been individually identified [20]. A genetic approach by coupling of mammalians 18S rRNA expansion segment ES9 into yeast ribosome also selectively recruits a set of transcripts containing internal ribosome entry site (IRES)-like element and facilitates cap-independent translation [21]. In the zebrafish model, replacement of maternal rRNAs with somatic rRNAs is observed during embryogenesis. This shifts from the preferential binding of 18S expansion segment ES3 over ES6 onto maternally expressed mRNAs to binding of ES6 over ES3 onto somatically expressed mRNAs in silico assay [22]. Other examples of rRNA sequence variants and/or modifications, or ribosomal protein paralogs, stoichiometry, and/or modifications that potentially contribute to ribosomal heterogeneity, have been recently reviewed by Li et al. [23] and Gay et al. [24]. The putative role of ribosomal heterogeneity in diseases or physiological protein biogenesis requires further investigation.

## 3. Detection of Ribosomal Heterogeneity

### 3.1. Chemical Probing of Ribosomal Components or rRNA Structures

Ribosomal proteins and rRNAs form complexes with other initiation and elongation partners, such as EIFs and tRNAs, to perform translational activities. To preserve the initial secondary or tertiary structures, crosslinking agents are commonly applied before subsequent sample processing. The following techniques in Table 1 have been used to identify partner molecules of ribosomal proteins and/or rRNAs. The psoralen and its chemical analogs intercalate into double-stranded nucleic acids and form covalent bonds, preferentially with pyrimidines, upon UV irradiation at 365 nm, in which the covalent bonds are broken or reversed by irradiation at 254 nm before subsequent enzymatic reactions [25]. The crosslinking targets of other chemicals or UV irradiation can be found in the corresponding references below. 

It is not possible to list every published technique in this study. Other chemicals to target specific sites of nucleic bases, ribose, and amino acids have been also applied to capture the interactions among RNAs and proteins. Readers are encouraged to check out some excellent review articles to acquaint other related methods and tips for further improvement [38,39,40,41,42,43].

### 3.2. Proximity Ligation to Generate rRNA-mRNA Chimeras for Sequencing

Proximity ligation has been widely used to determine RNA secondary structures by probing the RNA-RNA interactions, including the generation of rRNA-mRNA chimeras by joining the two RNAs from the same ribosome, as provided in some techniques listed in Table 1 [25,28,33,34,36]. Such techniques may potentially be used to identify individual rRNA sequence variants and corresponding mRNA substrates in a single-molecule RNA sequencing read. The information of the chimerical sequences can then be interrogated to detect specialized ribosomes, characterized by unique rRNA single-nucleotide and/or short-tandem repeat variants, along with their translating mRNAs. The longer the rRNA component of the chimera is obtained, the higher the chance is to detect rRNA sequence variants. The mRNA component only requires a short length for sequence identification. The protocol of proximity ligation may start with or without crosslinking followed by fragmentation or purification/enrichment before fragmentation, ligation or purification/enrichment before ligation, and additional round(s) of purification/enrichment before complementary DNA (cDNA) library preparation, and lastly next-generation high-throughput sequencing (next-gen Seq) plus bioinformatic analyses. The following methods listed in Table 2 are selected to represent diverse designs of protocols for generating chimeric RNAs, specific cDNA library, and polymerase chain reaction (PCR)-amplified double-stranded products for sequence analyses. The comparison below is intended to highlight the mechanism behind each step, not to create a preference list for the readers.

As exemplified above, the available proximity ligation approaches only decipher short chimeric sequences of up to a hundred nucleotides. They limit their applications to identify sequence variants across several hundred to thousands of nucleotides within the 18S, 28S, 5.8S, and 5S rRNAs. Close interactions between rRNAs and mRNAs have been observed in several organisms. The base-pairing of 5′leader of cap-dependent *Gtx* or *FGF2* mRNA to helix 26 of mouse 18S rRNA has been detected by sequence-specific translational assay or a masking assay with antisense oligonucleotides targeting the mRNA or rRNA [46]. A site-directed mutagenesis examination of the core IRES structure of the *IGF1R* mRNA shows a near-perfect Watson–Crick complementarity of the IRES to the G961 loop (helix 23b) of the human 18S rRNA in cultured cells [47]. A region around the AUG start codon of the mouse histone *H4* mRNA forms a folded structure next to the tip of, and a base-pairing with, the 18S rRNA helix 16 in rabbit ribosome, examined by cryo-electron microscopy (cryo-EM) [48]. Base-pairing of hepatitis C virus (HCV) IRES with the 18S rRNA helix 26 is also demonstrated in mouse cells expressing a recombinant 18S rRNA [49]. In a screening of RNA-RNA interactions in human cells, specific crosslinks of mRNAs to the 18S rRNA at helix 18 and 26 regions have been observed [33]. A direct binding of *Hoxa9* IRES-like element to expansion segment ES9S of the 18S rRNA is detected in ES9S-humanized yeast ribosome by cryo-EM [50]. Furthermore, the AUG codon-anticodon of an uncapped unstructured model mRNA has been localized to the vicinity of the 18S rRNA at nucleotides C1637 and U1191 with additional G1150 and G904 adjacent to position -1 and -3 of the mRNA, respectively [51]. Although SARS-CoV-2 also utilizes cap-dependent translation initiation, an IRES-like upstream opened reading frame (uORF) structure has been identified across 28 SARS-CoV-2 variants [52]. It appears that the 5′untranslated region (5′UTR) sequences of SARS-CoV-2 viral RNAs, even translated more efficiently than cellular mRNAs, do not protect translational inhibition by its NSP1 [53]. Evidence of direct contact between mRNAs and the 28S rRNA is still lacking but many sequence complementarities between the two have been predicted in silico [54]. The tRNAs may also bridge mRNAs to the 18S rRNA via their anticodon stem–loop or to the 28S rRNA via their D and T stem–loops [55]. 

The secondary structures of eukaryotic rRNAs, including yeast, mouse, and human 18S, 28S/5.8S, and 5S rRNAs, have been published [56,57,58]. Their stem–loops with corresponding nucleotides would assist in the interrogation of chimeric mRNA-rRNA sequences. In theory, 5′end of mRNAs containing proper hydroxyl or phosphate group can be ligated directly or via tRNAs to 3′ end of the 18S rRNA because interactions of mRNAs and/or tRNAs with the 18S rRNA have been repeatedly observed as exemplified above. The consistent base-pairing of 3′end of the 5.8S rRNA with 5′end of the 28S rRNA across species would allow proximity ligation to produce a 5.8S-28S chimera within species or in the recombinant ribosome. The direct interactions among 18S, 28S/5.8S, and 5S rRNAs, though not yet proven, may be promoted after the removal of ribosomal proteins. In addition, site-specific endonucleases or endo-exonucleases, to fragment mRNAs and/or rRNAs [59,60,61,62,63,64], may be useful to generate multiple free 5′- and 3′-ends for ligation. The mRNA-rRNA chimeras of hundreds or thousands of nucleotides in length may be directly interrogated by nanopore technology, described in the section below, or converted to long cDNAs by highly-processive reverse transcriptase before subsequent preparations that are compatible with long-read sequencing platforms. Alternatively, the long chimeras generated as RNAs or cDNAs may be separated individually, followed by fragmentation and library preparation with a unique barcoded adaptor for each chimera, proposed in the section below, before pooling together for pair-end reads of up to a hundred nucleotides by short-read sequencers.

### 3.3. Nanopore Gating of Individual Ribosomes

The estimated sizes of eukaryotic ribosomes range from 10 nm to 30 nm in diameter (yeast: about 15 nm; rat dorsal ganglia: about 24 nm; human: about 25 nm) with an estimated number of ribosomes per cell of 2 × 10^5^ in budding yeast, 5 × 10^5^ in fission yeast, and 4 × 10^6^ in HeLa cell [65,66,67]. The sucrose gradient ultracentrifugation, first developed in the 1960s [68,69], remains the mainstream technology to isolate ribosomes or their subunits. Other techniques, including affinity purification and size exclusion chromatography-ultrahigh pressure liquid chromatography, have also been used to shorten the time and steps of the isolation processes [70,71,72]. However, these methods obtain pooled ribosomes, or in some conditions, may separate monosomes from polysomes, and thus do not discriminate heterogeneous ribosomes. In order to determine the heterogeneity of ribosomes, individual ribosomes have to be separated before subsequent examinations of their rRNAs, RPs, mRNA substrate, and other translational components. One technology currently being explored as a proof-of-principle is the solid-state nanopore device.

A solid-state nanopore of 45 nm in diameter, etched and milled on SiO_2_/Si_3_N_4_ multilayer thin film as the component of a microfluidic chip, allows a single 50S ribosomal subunit to pass through the nanopore by controlling the voltage externally applied [73]. The same team subsequently constructed a programmable nanopore-optofluidic device, containing a nanopore drilled through SiO_2_/Ta_2_O_5_ layers, to deliver a single 70S ribosome across a 38 nm-wide nanopore. It also can deliver a single λ-DNA across a 20 nm-wide nanopore with a voltage feedback control for the selected number of biomolecules to translocate via the nanopore [74]. A quartz-based glass nano-pipette of ≈60 nm in diameter, produced via laser pulling, has been used to discriminate individual 80S ribosomes from polysomes according to voltage peak amplitude and particle dwell time [75]. These nanopore technologies provide great potential to obtain individual ribosomes for subsequent RNA and protein characterizations.

### 3.4. Nanopore RNA Sequencing and/or Structural Analyses

As discussed above, rRNA sequence variations and post-transcriptional modifications, as well as engagement of tRNAs, contribute to the heterogeneity of ribosomes. A long-read sequencing platform is needed to interrogate hundreds to thousands of nucleotides in length. The nanopore technology provides such long-read capability and direct RNA sequencing without cDNA synthesis and PCR amplification steps, which are required in short-read sequencing protocols. The Oxford Nanopore Technologies (ONT) first demonstrates a direct RNA read length of over 1500-nucleotide yeast transcripts with >90% accuracy and with the ability to detect RNA modifications, including N6-methyladenosine (m6A) and 5-methylcytosine (5-mC), using the MinION R9.4 flowcell [76]. The ONT utilizes an array of protein nanopores and provides kits and software to conduct the entire sequencing process. The newer R9.4.1 flowcell has been used to identify pseudouridine (Ψ), an isomer of uridine (U), in SARS-CoV-2 RNA by analyzing ionic current and dwell time data [77]. A full-length of ≈1.5 kb *E. coli* 16S rRNA sequence, 7-methylguanosine and pseudouridine modifications, and single nucleotide variants among 7 rRNA copies have been also detected using FLO-MIN106 SpotON flowcell from the ONT [78]. A protocol to sequence human 18S, 28S, 5,8S, and 5S rRNAs based on the MinION platform was also developed to produce a high-sequencing output of over four thousand sequence reads on each rRNA [79].

In addition to sequencing analyses, RNA secondary or tertiary structures with or without chemical modifications are also detectable by nanopore technology. The RNA structure analysis using the nanopore sequencing (abbreviated as PORE-cupine) method, which applies the MinION flowcell to sequence chemically modified RNAs, demonstrated the feasibility to reproduce RNA secondary structures, including protein-binding sites from established short-read sequencing analyses of RNAs modified by the same chemicals [80]. This PORE-cupine method is also able to decipher the structures of thousands of coding genes, noncoding genes (including four cytosolic rRNAs), and pseudogenes along with the identification of two or more single-nucleotide variants in 90 transcripts across the hESC transcriptome. This provides a fast global view of RNA-RNA and RNA-protein interactions. Methodologies capable of capturing native tertiary RNA structures have been explored by other nanopore technologies using different pore systems. A Si_3_N_4_-nanopore of ≈3 nm in diameter was used to differentiate 5 tRNA species, which are critical partners for ensuring translational fidelity by ribosome. This is according to characteristic dwell time and current blockade level after analyses by an optimized machine learning algorithm [81]. Modeling of pseudoknot folding kinetics was carried out for a known RNA sequence captured on an α-hemolysin protein nanopore [82], potentially for validating the impact of sequence variant to RNA tertiary structure. The dual characteristics of a large vestibule (≈4.8 nm) and the narrow constriction (≈1.2 nm) of a *Mycobacterium smegmatis* porin A (MspA) have been utilized to construct a nanopore for determinations of RNA tertiary structure and sequence in a single molecule through trapping and translocation [83]. The MspA-nanopore can make a distinction among microRNA (22-nt double strand), small interfering RNA (21-nt double strand), tRNA (76 nt), and 5S rRNA (120 nt) based on electric current fluctuations between trapping and translocation processed by a machine learning algorithm. A 23S rRNA (2904 nt) and a 16S rRNA (1542 nt) have been also tested in this nanopore system but their trapping and translocation characteristics are less defined and require further optimization.

### 3.5. Single-ribosome Mass Spectrometry

The possibility to identify individual ribosomal proteins, some containing post-translational modifications, of an intact ribosome by mass spectrometry has been demonstrated in *E. coli* 70S ribosome, and its 30S and 50S subunits, through a controlled dissociation by manipulating the energy of gas-phase collision [84]. However, the broadening of mass/charge (*m*/*z*) peaks, likely contributed by ions from the buffer solution and protein-free RNA, affects the accuracy of ribosome mass determination and the resolution of individual ribosomal proteins. An ultra-high mass range spectrometry, developed to enhance the detection of high *m*/*z* ions, improves the accuracies of mass measurements for the intact or native *E. coli* 70S, 30S, and 50S ribosomes. It further identifies multiple populations of a 30S or 50S subunit as a result of stoichiometric differences in ribosomal proteins and/or the presence of transient ribosome-interacting proteins, confirming the notion of ribosomal heterogeneity [85]. The same research group also incorporated charge detection mass spectrometry (CDMS) into the commercial Orbitrap mass analyzer to simultaneously determine the z and *m*/*z* of individual ions. It also demonstrated a high accuracy of assigning masses to native human 80S, 40S, and 60S ribosomes and high resolution for detecting heterogeneity of ribosomes using the Orbitrap-based CDMS [86]. Since mass spectrometry requires a sufficient number (for example, 10^3^ to 10^5^) of single ion events to obtain overlapping series of peaks, the ribosome concentrations of 0.1 to 5 μM (equivalent to about 6 × 10^16^ to 3 × 10^18^ ribosomes) used in the above mass spectrometry methodologies add more layers of complexity to differentiate heterogeneous ribosomes from a large pool of ribosomes.

### 3.6. Microfluidic Droplets for Separating Ribosomes or Indexing rRNAs/mRNAs

Since the heterogeneity of ribosomes arises from the individuality of each ribosome or ribosomal population, examinations of a large number of translating ribosomes separately may provide a solution for matching a sequence signature of the mRNA or translated peptide to a specific ribosome or ribosomal population. To provide such a solution, several microfluidic devices, capable of sorting functionalized magnetic beads or generating droplets via micrometer-scale channels to encapsulate enzymatic reagents, are discussed or proposed. One microfluidic device has been conceptualized to lyse cells in the microfluidic channel, followed by capturing translating ribosomes by magnetic particles covalently attached with oligonucleotides. This is complementary to a specific mRNA or attached with antibodies against a specific ribosomal protein, and separating as well as sorting the bound ribosomes for downstream analyses [87]. A microfluidic capillary device has been manufactured to generate water-oil-water, double-emulsion droplets that encapsulate ribosomes and reagents for translating a membrane protein inside the droplets [88]. Ligation of cDNAs with unique adaptors containing a specific sequence barcode or index inside a droplet is achievable in many commercially available microfluidic droplet generators. A single-cell combinatorial fluidic indexing (scifi) is further developed to maximize the usage of reagents [89]. Each droplet houses unique adaptors and encapsulates one or a few pre-indexed cells or nuclei while traveling through the micrometer channel of the microfluidic droplet generator. Inside the droplet, cDNAs, which are synthesized in permeabilized cells or nuclei using a pre-indexed primer, are released from the single or the few cells or nuclei and are subsequently ligated with the barcoded or indexed adaptors. Thousands to millions of cells or nuclei are processed enzymatically inside corresponding thousands to millions of droplets. Barcode-tagged cDNAs are then pooled for library preparation and high-throughput sequencing. The sequencing data from the same barcode represent a single-cell RNA profile. In theory, the cells or nuclei can be substituted with ribosomes and the scifi protocol can be modified to ligate rRNAs and mRNAs with unique adaptors for each ribosome inside a droplet and to sequence a pool of barcoded rRNAs and mRNAs. In this case, rRNAs and corresponding mRNA substrate from the same translating ribosome would be identified by the presence of the same barcode.

## 4. Final Notes

**Figure 1:**
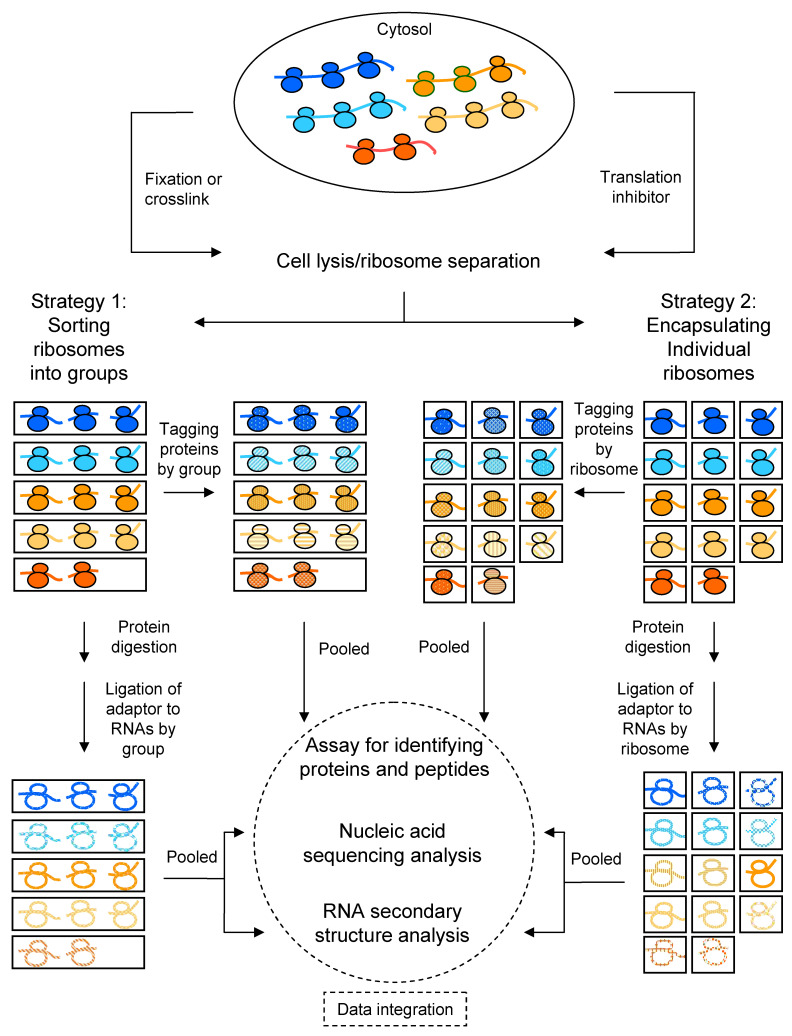
A general strategy for developing a protocol to characterize heterogeneous ribosomes by group or by ribosome. Heterogeneous ribosomes are colored differently, and active ribosomes, containing their corresponding mRNAs and/or translated peptides, can be separated by sorting into groups or by encapsulating individual ribosomes. The sorting may be achieved by solid-phase isolation according to unique protein and/or RNA properties, such as specific ribosomal proteins, translational cofactors, mRNAs, and translated peptides. This would be followed by transferring the solid-phase captured ribosomes into different compartments of a micro- or nano-plate. Nanopore-equipped, microfluidic, droplet-generating devices are suitable for encapsulating ribosomes individually. Protein or peptide components of the ribosomes can be tagged using reactions, such as by click chemistry [90], by ribosome group after sorting, or by an individual ribosome after encapsulation. Tagged ribosomes are then pooled for protein analyses, such as mass spectrometry, to obtain the identities of protein or peptide components, and the origins of heterogeneous ribosomes can be traced after decoding their unique tags. An aliquot of the sorted ribosomes from the same compartment or of the encapsulated ribosomes can be processed, for example, by extraction or protein digestion, to acquire suitable RNAs for subsequent adaptor ligation. The ligation step may also generate rRNA-mRNA chimeras that are useful for analyses of RNA secondary structures and validation of rRNA-mRNA pairs. For crosslinked RNAs, a reversal step is required to break the crosslink before downstream enzymatic steps in preparation of RNAs, cDNAs, or PCR-amplified products for nucleic acid sequencing. Each adaptor contains a unique barcode or index sequence that is used to identify the origin of rRNAs and mRNAs within the same group of or within the individual ribosome. The identities of translated peptides from protein analyses can then be integrated with mRNA sequence analyses to find the origins of the groups of or individual active ribosomes and to differentiate heterogeneous ribosomes.

Several strategies to differentiate heterogeneous ribosomes, or to identify specialized ribosomes with their mRNA substrates, are discussed to assist in developing assays for clinical study. The above-exemplified methodologies do not appear to be readily applicable to or to be currently used in the testing of clinical samples. To determine the role of ribosomal heterogeneity in COVID-19-related pathological characteristics, examination of clinical samples is needed. The key obstacle of converting research tools for clinical use is the capability to analyze a tiny amount of patients’ samples. Preferably, an assay should be able to obtain test results from a clinical sample with a quantity at nano- or micro-scale. Since the respiratory system is the major route of SARS-CoV-2 viral entry, nasal or nasopharyngeal swab, or bronchial lavage, is commonly performed. It would provide a sufficient number of ribosomes, estimated to be millions per cell, for assays that have a sensitivity to detect a single ribosome. Chemical or UV fixation is required for samples collected from COVID-19 patients to inactivate the virus. Some crosslinking agents described in the chemical probing section above have been routinely used to fix cells or tissues in the clinical laboratory. Samples from healthy patients may not require fixation if they are immediately analyzed. Integration of different technologies into a central location or a community of close vicinity would facilitate the sample processing and data collection plus computation. Careful design of a clinical trial to recruit a sufficient number of participants is essential to reach statistical power in multivariate analyses. Comorbidity of COVID-19 with chronic diseases is a frequent clinical manifestation in severe cases. The study of ribosomal heterogeneity in COVID-19 patients may also shed light on other diseases. The process to develop a clinical protocol for examining the role of ribosomal heterogeneity in COVID-19 patients is a challenging task and requires joint efforts from multidisciplinary areas. A general strategy is proposed here, and a schematic approach is outlined in Figure 1. This article is intended to foster discussions among researchers and clinicians for a better understanding of the current pandemic or better preparation for the next pandemic. The assays to identify specialized ribosomes, along with their corresponding mRNA transcripts and/or peptides, are also applicable to study other diseases.

## Figures and Tables

**Table 1:** Chemical and/or physical probing for interactions among RNAs and proteins.

Technique	Chemical Probe	Irradiation	Probing Structure ^a^	Ref.
SPLASH (Sequencing of psoralen crosslinked, ligated, and selected hybrids)	Psoralen-PEG3-Biotin	UV-A (365 nm)	rRNA-rRNA, mRNA-rRNA, snRNA-rRNA, snoRNA-rRNA, and mRNA-mRNA	[25]
COMRADES (Cross-linking of matched RNAs and deep sequencing)	Psoralen-triethylene glycol azide	UV-A (365 nm)	rRNA-rRNA and interaction of viral RNA with cellular RNAs	[26]
SHAPE-JuMP (Selective 2′-hydroxyl acylation, primer extension and juxtaposed merged pairs)	Trans bis-isatoic anhydride		RNA-RNA	[27]
LIGR-seq (Ligation of interacting RNA and high-throughput Sequencing)	4′-aminomethyltrioxalen (AMT)	UV-A (365 nm)	snRNA-snRNA, snoRNA-mRNA, and rRNA-rRNA	[28]
hiCLIP (RNA hybrid, individual-nucleotide resolution, UV crosslinking and immunoprecipitation	Formaldehyde	UV-C (254 nm)	Protein-protein and RNA-protein RNA-protein	[29]
XL-MS (Crosslinking/ mass spectrometry)	disuccinimidyl diacetic urea		Protein-protein	[30]
PTex (Phenol-Toluol extraction) crosslinked RNA-protein	With/without 2-iminothiolane	UV-C (254 nm)	RNA-protein	[31]
Chemically Reversible Acylation	bis-nicotinic azide reversible interaction		RNA-RNA	[32]
PARIS 2 (Psoralen analysis of RNA interactions and structures, second version)	amotosalen or 4′-aminomethyl trioxalen	UV-A (365 nm)	rRNA-rRNA, mRNA-rRNA, and snoRNA-rRNA	[33]
CLASH (Crosslinking, ligation, and sequencing of hybrids)		UV-C (254 nm)	snoRNA-rRNA and RNA-protein	[34]
XRNAX (Protein-Crosslinked RNA Extraction)		UV-C (254 nm)	RNA-protein	[35]
RIC-seq (RNA in situ conformational sequencing)	Formaldehyde		rRNA-rRNA, snoRNA-rRNA, rRNA-mRNA, and snoRNA-mRNA	[36]
In situ CLMS (In situ cross-linking and mass spectrometry)	Formaldehyde Disuccinimidyl suberate		Protein-protein Protein-protein	[37]

^a^ snRNA: small nuclear RNA; snoRNA: small nucleolar RNA.

**Table 2:** Comparison of selected proximity ligation protocols.

Method	Crosslink (for Purification or Enrichment)	Fragmentation (for Purification or Enrichment)	Ligation (for Purification or Enrichment)	Library Preparation and Sequencing	Cautions ^a^	Ref.
SPLASH	Psoralen-PEG3-Biotin/UV-A (Trizol RNA extraction)	MgCl_2_, pH 8.3, 95 ^°^C (urea gel to obtain 90–110 nucleotides [nts] followed by streptavidin bead purification)	T4 PNKinase followed by T4 RNA ligase I (Trizol)	Crosslink-reversal by UV-C, 3′adaptor ligation, urea gel to obtain 110–130 nts, cDNA synthesis and circularization, PCR (200–300 bp), and next-gen Seq	Low cell permeability of biopsoralen; UV-C damage to RNAs	[25]
CLASH	UV-C (IgG bead RNAs-protein complex purification after cell lysis)	RNase A and T1 single-stranded endo-RNase (Nickel bead to bind His-tag snoRNP)	On-bead 3′-phosphate removal, followed by 3′ and 5′ adaptor ligation by T4 RNA ligase (gel isolation of the RNA-protein complex)	Proteinase K digestion, RNA extraction, cDNA synthesis, PCR (>60 bp), TA cloning, next-gen Seq	Detection of interaction for a specific protein	[34,44]
RPL (RNA proximity ligation)	None	Endogenous RNase for yeast cell; RNase A and T1 for human cell	T4 PNKinase followed by T4 RNA ligase I (Trizol)	Modified Illumina TruSeq RNA kit (RNA fragmentation, cDNA synthesis, and PCR) and pair-end 80/101-bp reads in a next-gen Seq	Low abundance of mRNAs	[45]
LIGR-seq	AMT/UV-A (Trizol/DNase I treatment)	S1 endonuclease after rRNA depletion (phenol-chloroform extraction)	circRNA ligase (3′-5′ exo-RNase R digestion followed by phenol and chloroform extraction)	Crosslink-reversal by UV-C, precipitation of RNAs, modified Clontech SMARTER library prep, gel isolation of >200 bp products for next-gen Seq	Reduction of rRNA due to depletion step	[28]
COMRADES	Psoralen-triethylene glycol azide/UV-A (Qiagen RNeasy lysis/purification, followed by on-bead isolation with antisense probes)	RNase III db-stranded endo-RNase (Zymo RNA Concentrator, chemical linking of biotin-alkyne to azide on crosslinked RNAs and on-bead isolation)	RNA ligase 1 (Zymo RNA Clean and Concentrator)	Crosslink-reversal by UV-C, 5′- and 3′-adaptors ligation, cDNA synthesis, PCR, and on-gel size selection for pair-end 150-bp reads in a next-gen Seq	Detection of interaction for a specific RNA	[12]
RIC-seq	Formaldehyde (quenched, washed, and cell permeabilization)	In-cell micrococcal endo-exonuclease digestion (cell wash, 3′dephosphorylation of fragmented RNAs, ligation of pCp-biotin, removal of 3′phosphate of pCp-ligated RNAs, cell wash, 5′phosphorylation, and cell wash)	In-cell T4 RNA ligase ligation (cell wash and lysis by proteinase K, Trizol extraction, DNase I treated, fragmentation of RNA in MgCl_2_ at 94 ^°^C, on-bead enrichment of biotin-labeled RNA chimeras, and phenol and chloroform extraction)	Strand-specific cDNA synthesis (hexamers to generate the first strand, RNase H-truncated RNA strand as primer to yield a dUTP-containing second strand, 3′adenylation, ligation of 3′ and 5′adaptors, and digestion of the dUTP strand), PCR and on-gel isolation of 200–450 bp products for next-gen Seq with pair-end reads of about 260 bp	Enriched interactions: mRNA-mRNA > ncRNA-ncRNA > mRNA-ncRNA > pseudogene- pseudogene (ncRNA includes lncRNA, snoRNA, and eRNA)	[36]
PARIS2	Water-soluble amotosalen/UV-A (cell or tissue wash, cell lysis with Guanidine Isothiocyanate followed by Proteinase K and DNase digestions with phenol/isopropanol extraction after each digestion)	ShortCut RNase III double-stranded endoribonuclease (RNA precipitation and 2D urea-gel to isolate RNA fragments of 50 nts or higher)	T4 RNA ligase 1 (RNA precipitation)	Crosslink-reversal with little RNA damage (UV-C plus acridine orange singlet quencher), 3′adaptor ligation, SuperScript-IV to generate cDNAs, RNase H/A/T1 to remove RNAs, CircLigase™ II to circularize cDNAs, PCR, and on-gel isolation of 175 bp or higher for next-gen Seq	Low efficiency of proximity ligation by T4 RNA ligase (≈10% gapped or chimeric reads); bias toward uridine crosslink by AMT or amotosalen	[33]

^a^ lncRNA: long-noncoding RNA; snoRNA: small nucleolar RNA; eRNA: enhancer RNA.

## Data Availability

Not applicable.
